# Multi-label classification of research articles using Word2Vec and identification of similarity threshold

**DOI:** 10.1038/s41598-021-01460-7

**Published:** 2021-11-09

**Authors:** Ghulam Mustafa, Muhammad Usman, Lisu Yu, Muhammad Tanvir afzal, Muhammad Sulaiman, Abdul Shahid

**Affiliations:** 1 Department of Computer Science, Capital University of Science and Technology, Islamabad, 44000 Pakistan; 2 Department of Computer Science, The National University of Computer and Emerging Sciences (FAST), Islamabad, 44000 Pakistan; 3 Present Address: School of Information Engineering, Nanchang University, Nanchang, 330031 Jiangxi People’s Republic of China; 4 State Key Laboratory of Computer Architecture, Institute of Computing Technology, Chinese Academy of Sciences, Beijing, 100190 China; 5 Department of Computer Science, Namal Institute, Islamabad, 42200 Pakistan; 6 Institute of Computing, Kohat University of Science and Technology, Kohat, 26000 Pakistan

**Keywords:** Computer science, Scientific data

## Abstract

Every year, around 28,100 journals publish 2.5 million research publications. Search engines, digital libraries, and citation indexes are used extensively to search these publications. When a user submits a query, it generates a large number of documents among which just a few are relevant. Due to inadequate indexing, the resultant documents are largely unstructured. Publicly known systems mostly index the research papers using keywords rather than using subject hierarchy. Numerous methods reported for performing single-label classification (SLC) or multi-label classification (MLC) are based on content and metadata features. Content-based techniques offer higher outcomes due to the extreme richness of features. But the drawback of content-based techniques is the unavailability of full text in most cases. The use of metadata-based parameters, such as title, keywords, and general terms, acts as an alternative to content. However, existing metadata-based techniques indicate low accuracy due to the use of traditional statistical measures to express textual properties in quantitative form, such as BOW, TF, and TFIDF. These measures may not establish the semantic context of the words. The existing MLC techniques require a specified threshold value to map articles into predetermined categories for which domain knowledge is necessary. The objective of this paper is to get over the limitations of SLC and MLC techniques. To capture the semantic and contextual information of words, the suggested approach leverages the Word2Vec paradigm for textual representation. The suggested model determines threshold values using rigorous data analysis, obviating the necessity for domain expertise. Experimentation is carried out on two datasets from the field of computer science (JUCS and ACM). In comparison to current state-of-the-art methodologies, the proposed model performed well. Experiments yielded average accuracy of 0.86 and 0.84 for JUCS and ACM for SLC, and 0.81 and 0.80 for JUCS and ACM for MLC. On both datasets, the proposed SLC model improved the accuracy up to 4%, while the proposed MLC model increased the accuracy up to 3%.

## Introduction

Larsen and Von^1^ claim that every five years, the number of research articles doubles. The scholarly article creation process has never been interrupted; rather, it has accelerated day by day^2^. In 2015 Ware and Mabe^3^, published that about 28,100 journals generate 2.5 million research articles each year. The search engines cannot properly categorize or index these research papers based on their content. The performance of the search engines can be improved if the articles are tagged to their relevant domains. This massive disarray of research articles drew the attention of a large research community, who demanded that the publications be classified into their proper categories. The researchers concentrated on classifying the documents in such a way that maximal and relevant information could be retrieved^4^. Due to the vast data available on the internet, researchers face difficulty in classifying articles into acceptable categories.

Several machine learning algorithms are being used to categorize the documents efficiently^5–7^. These approaches addressed the problem of research articles classification^4,8^. Every research paper is classified into one or more categories. The issue of mapping research articles with associated categories can help scholars in a variety of ways, including (1) assisting researchers in finding relevant materials to their topic, (2) Locating appropriate literature to explain the proposed study’s background concept, and (3) For user inquiries, search engines and digital libraries return appropriate documents. The classification of research publications is primarily separated into two categories: (1) content-based approaches and (2) metadata-based techniques.

Because of the diversity of features, content-based approaches typically give better results than metadata-based techniques^9–11^. However, one of the most significant disadvantages of content-based techniques is the unavailability of the most of articles publicly. As is the case with some re-known journals like ACM and IEEE have not made the entire articles publicly available. In such scenarios, some scholars have turned to metadata as an alternate method of categorizing research papers^12–14^. Metadata of the research articles like title, keywords, key terms, authors, and categories are freely available online. This work mainly focuses on research document classification in the Computer Science domain. The proposed model addresses the classification issue by using metadata parameters individually as well as in combination. Each metadata parameter holds significant potential and their collective contribution can beneficiate in improving the accuracy.

One of the most fundamental issues in text mining and information retrieval is text representation (IR)^15^. The goal of text representation is to numerically convert unstructured text data into mathematically quantifiable documents. The current state-of-the-art approaches use the traditional statistical measures such as Term Frequency (TF), Bag of Word (BOW), and Term Frequency and Inverse Document Frequency (TFIDF)^9–14^. As a result, they have overlooked the semantic and contextual information of keywords, potentially leading to the incorrect categorization of research publications. In this study, one of the most well-known techniques, word embedding, is used^16–18^. It can recognize the context of words in a document, such as semantic similarity, grammatical similarity, and relationships with other words. Word2Vec, one of the most prominent techniques for learning word embedding using shallow neural networks, is employed in this study. It was created by Mikolov et al. at Google in 2013^19^.

In the present state-of-the-art^12–14^, researchers first chose the strategy of asking domain experts for similarity threshold values or setting arbitrary values and then ensure it on the dataset through trial and error, which is a time-consuming operation. Dependence on domain specialists or arbitrary values is insufficient for the goal. The current literature identifies several strategies for automating the classification of scientific research papers into predetermined categories. The noted research gap is that most of the studies have relied on traditional statistical measures to quantify the similarity between textual sources. For textual representation, they merely recorded the data based on frequency rather than the meanings and context of phrases. In this study, multi-label classification is utilized to give several labels to documents based on some similarity threshold values, which serve as the bottom limit for categorizing research articles. The average similarity score of a test document of each category is compared to the similarity threshold value for that category. The categories with a score higher than the threshold value are chosen as the test document’s final category. The goal of this study is to see how much a semantic model can increase classification accuracy when compared to a statistical measure of individual and combined Metadata features and how can we set a multi-label categorization threshold value.

## Literature

When the first document classification strategy was proposed by the scientific community in the 18th century, the process began in several branches, and as a result, the research community’s focus shifted to the categorization of a certain type of document, such as (1) newspapers. (2) Webpages, and so on^20–22^. Due to rapid invention in literature, the research community’s focus shifted to research paper classification. The proposed approaches in the literature that are currently state-of-the-art can be classified into two major categories: content-based approaches and metadata-based approaches.

### Content based approach

Content based approaches depend on content of the research articles. In 2015, Le et al.^23^ performed survey on all existing feature selection approaches for text classification. In this survey, they discussed all method of feature selection and feature reduction. They categorized all the method into two broad categories (1) wrapper (2) filter. Performance of filter method is significantly better than wrapper method because filter does not depend on classification algorithm. In literature mostly researchers used the filter technique for text classification.

In 2016, Tang et al.^22^ proposed Bayesian classification approach for text classification by analyzing specific features for each class instead of using global features for all classes. They built rules for classification by using Baggenstoss’s PDF Project Theorem for each specific class features. In 2016, Zhou et al.^24^ proposed a content-based approach using naive Bayes and Logistic regression algorithms. They used two diversified datasets from computer science domain which have already annotated such as: (1) CiteSeerX, (2) arXiv. The concluded achieved F1 Score on arXiv and CiteSeerX datasets are 0.95 and 0.75 respectively. In 2015, Zhong et al.^25^ proposed semantic similarity on different features for classification of text. Experiment is performed on two different datasets such as (1) Routers-10 (2) 20-Newsgroups. They conducted a series of experiment on the Routers-10 and 20-Newsgroups dataset and apply Support Vector Machine algorithm (SVM) by achieving F-Score of 0.76 for 20-Newsgroups and 0.91 for Router datasets. In 2012, Chekima et al.^26^ proposed document categorizer agent, based on Naive Bayes Classifier. After performing experiment on 1000 Computer Science papers, 91% accuracy is obtained. Cai and Hofmann^27^ proposed an approach to classify text documents based on SVM classifier. This approach has been evaluated using WIPO-alpha Collection dataset. Another approach of hierarchical multi-label text classification has been proposed by Baker and Korhonen^28^, in which neural network model is used for classification. The results have been evaluated by using biomedical field data. The results conclude that document level classification performs better than sentence level classification.

In 2008, Kannan and Ramaraj^9^, developed a system for text classification based on similarity of text. In this approach feature selection framework has been presented in which Information Gain (IG) Score is used for every word to perform text classification. Authors have also presented the initial learning model; in which unlabeled document has been randomly selected and annotated by field experts. This approach has been tested on Reuter dataset which contains almost 21578 documents. After conducting extensive experiment, it is identified, that on sample of 2000 document their approach attained improved value of F-Measure 0.90. Moreover, the outcome of the study also reported that by reducing vocabulary size, the rate of classification increases.

One of another content-based approach is also proposed by Santos and Rodrigues^10^. This approach comprised of two main steps, (1) Create a dataset of a document in the form of multi-label hierarchy, these documents were extracted from ACM digital library. (2) Developed a model for multi-label text classification by combining various classification algorithms. This approach utilized title, abstract and keywords as a feature for multi-label document classification. This approach also utilized different classification algorithm, like Binary relevance, Label Power set, sequential minimal optimization and Naive Bayes Multinomial etc. After conducting comprehensive experiment, the results revealed that Binary relevance combined with Naive Bayes Multinomial perform extraordinary and achieve 0.88 f-measure as compared to others classifier they used individually as well as combined.

Jindal and Shweta^29^, proposed a method for Efficient Multi-label Text categorization of the research articles. This approach used the concept of lexical and semantics analysis to solve the problem of multi-label categorization of text documents. In lexical analysis step, tokens have been identified from research articles based on IEEE taxonomy. In semantic analysis step, relationships between the tokens are analyzed using the standard lexical database of words, i.e. WordNet. In next step, classification is performed, in which classes of tokens are determined using IEEE taxonomy. This approach is evaluated on 150 papers of computer Science domain. The outcome of the study revealed that their approach achieved accuracy up to 0.75.

### Metadata-based approach

The existing metadata-based approaches uses metadata of research articles for classification of research document task. Metadata of research document includes title, author, keywords, general terms, categories etc. This type of metadata is almost freely available, while the whole content of the data is not freely available online. So that is the big motivation for the research community to move from content to freely available metadata of the research documents.

Yohan et al.^30^ proposed a technique using natural language processing for finding name entities and classified them in their respective categories. The approach has comprehensively been evaluated using different Newspaper and Telugu wiki datasets. This approach concludes precisions in range of 0.79 to 0.94.For improvement in classification, Zhang^12^proposed a model based on structural and citation-based information. In this approach, they combine the structural information (title, abstract) with citation of research paper for some big achievement in document classification. Sajid et al.^14^ proposed fuzzy logic-based classifier for the classification of research paper in Computer Science domain. For experimental purpose they select the JUCS datasets due to the coverage of all areas of Computer Science domain. After performing detailed evaluation of the approach, the results revealed that the approach achieved 0.93 precision and 0.96 F measure for single label classification measures.

For document classification, another metadata extraction approach is proposed by Flynn^13^. This approach proposed the “post hoc” system for categorizing the documents. This approach is divided into two phases. (1) In first phase, they extracted metadata based on template, (2) In second phase, they have performed classification based on these extracted metadata. For an evaluation purpose, diversified dataset of defense technical information center (DTIC) is used, which contain one million data of scientific articles, PHD thesis, research papers of conferences, journals, slides and law document etc. The results revealed that this approach predict the category of document 0.83 time correctly.

In another study, Bayesian based approach has been presented by khor and Ting^31^ to classify research papers. In this study, 400 research papers from education conference have been considered as a dataset and mapped to four different classes including e-learning, cognition issues, teacher instruction and intelligent coaching system. The researchers contended that there are keywords traits that can be used for categorizing the papers. This approach used a features selection algorithm to extract the keywords related to each topic. This approach is solely based on keywords-based features.

Ali and Asghar^32^, proposed multi-label scientific document classification based on metadata features. This approach utilized two metadata features (title and keywords). For performing multi-label classification, data is prepared for single label classification by using four different conversion techniques (Min, Max, Ran, and Single). This approach also used different similarity measures for finding the relevancy between documents and labels. This approach utilizes PSO based classifier for the classification of documents. This approach is evaluated on two different dataset of research articles (JUCS and ACM). The outcome of the study revealed that their approach achieved accuracy up to 0.78.

## Methods

**Figure 1:**
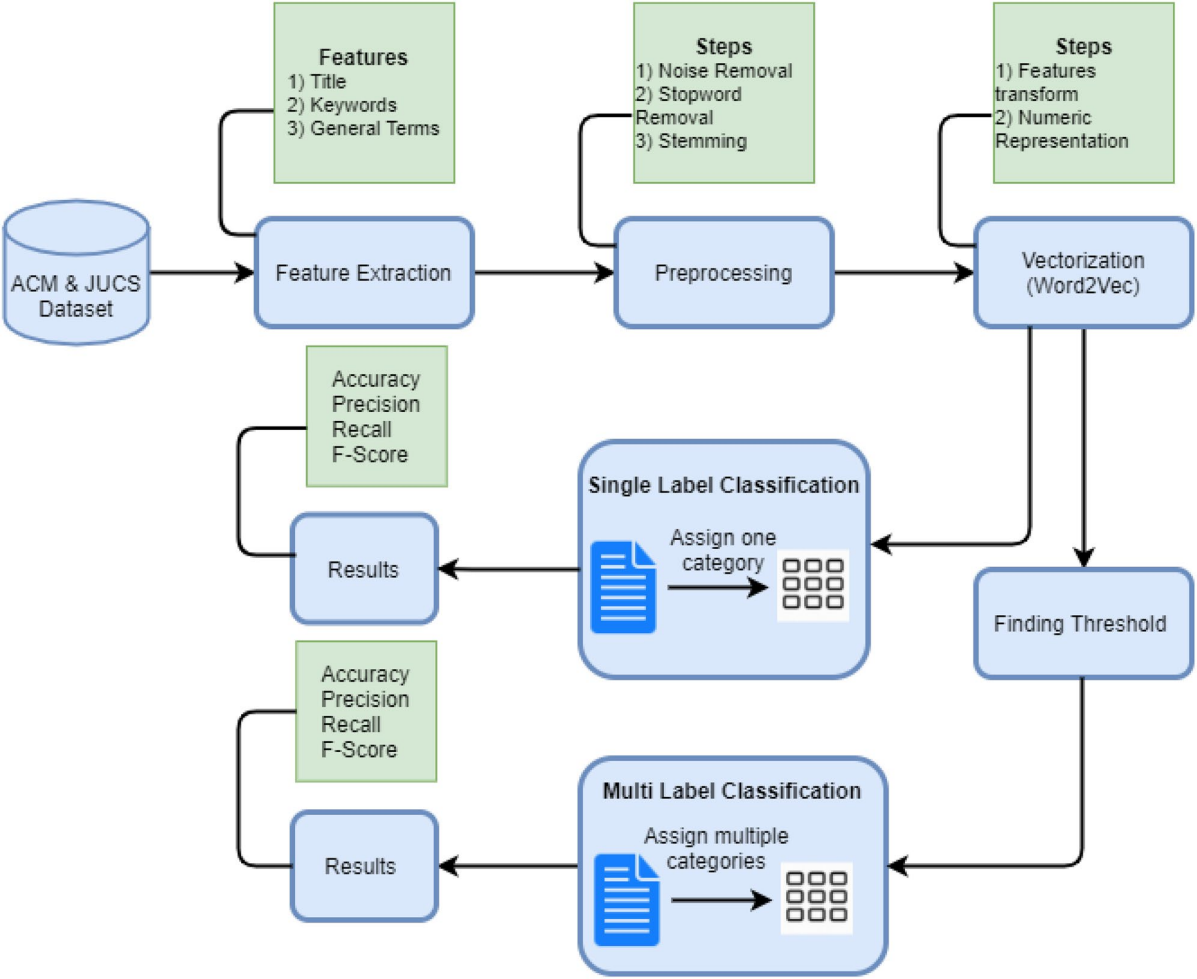
Architecture diagram.

After critical analysis of already proposed approaches delineates that the research article classification community has proposed different techniques to classify the research articles into single and multiple categories. The motivation of proposed work from literature review is: (1) none of the literature study comprehensively evaluate the freely available metadata individually and its possible combination, (2) there does not exist any study that utilize semantic model for text representation and consider both context and semantic term, (3) In case of multi-label classification, no study exist which has identified the threshold value by rigorous analysis of data. These observations led us to propose a technique to address the issues discuss above. The proposed framework performed single and multi-label classification of research articles into predefined ACM hierarchy by comprehensively evaluating the metadata features (individually as well as its combination). In this section, the proposed methodology is described for the classification of research articles. The Fig. 1, represent the architecture diagram of our proposed technique.

### Dataset

To comprehensively evaluate the proposed system, one needs to carefully select the dataset. To evaluate the proposed framework, we have carefully picked two best suited diversified datasets. One of them is based on research publications from Journal of Universal Computer Science (J.UCS)^33^ and another one contains research publications from the Association of Computing Machinery (ACM) and developed by Santos et al.^10^. The reason for the selection of J.UCS dataset is twofold: J.UCS covers all topics of Computer Science and the researchers who published their work belong to diversified domains and geographical regions, which can help us to perform comprehensive evaluation. Similarly, the reason for the selection of ACM dataset is that it contains research publications from different conferences, journals and the workshops. J.UCS dataset contains 1,460 research publications. It has extended the ACM CCS98 with two more classes like L and M. Therefore, at top level, there are 13 distinct classes in J.UCS dataset rather than 11 classes as per ACM classification (i.e. classes A-K correspond to the ACM classification with its sub classifications, classes L (Science and Technology of Learning) and M (Knowledge Management) were added to reflect the development of the Computer Science discipline). However, we have selected top 11 categories from both datasets. Moreover, ACM dataset built by Santos^10^ contains 86,116 research publications from conferences, journals and workshops of diversified domains. Both datasets have significant numbers of research articles associated with multiple classes. The detailed statistics of both data sets are presented in Table 1.

**Table 1:** Dataset statistics.

Features	JUCS dataset	ACM dataset
Total number of research papers	1460	86,116
Total number of journals or conferences or workshops	1	2240
Single-label research papers percentage	51(%)	54(%)
Multi-label research papers percentage	49(%)	46(%)
Total number of classes or categories at root level	13 (11 Selected)	11
Name of metadata of research paper	Title, Keyword, Categories	Title, Keyword, General Terms, Author name and Categories
Number of records of A to K categories are	A(35), B(45), C(123), D(311), E(55), F(302), G(110), H(380), I(235), J(86), K(149)	A(644), B(5723), C(8735), D(17628), E(539), F(6257), G(3616), H(17845), I(15099), J(1343), K(9908)

### Feature extraction and combination

All the possible combinations of the metadata like Title, keywords, General Terms and categories are selected from the both JUCS and ACM dataset. The selection of specific metadata’s as a feature is based on the following reasons:

1. The title of paper holds potential terms that can assist in determining the category of research article.
2. Keywords and general terms are explicitly assigned by the actual authors of papers that are mostly from relevant areas.

**Figure 2:**
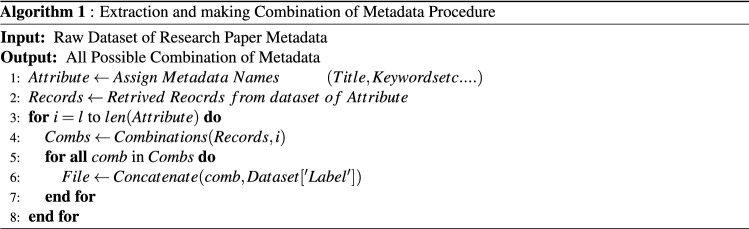
Generating possible combination.

From JUCS dataset we have selected two metadata such as: (1) Title and (2) Keywords due to free availability of these metadata in JUCS dataset. Similarly, from ACM dataset we have selected three metadata such as: (1) Title (2) Keywords and (3) General Terms. Afterwards to comprehensively evaluate all the metadata features we have formed all the possible combinations (presented in Table 2) of these metadata features of both datasets by using Algorithm 1 (presented in Fig. 2).

**Table 2:** Possible combination.

Datasets	Uni features	Bi features	Tri features
JUCS dataset	(1) Title	1) Title and keywords	No tri features
	(2) Keywords		
ACM dataset	(1) Title	(1) Title and keywords	(1) Title, keywords and general terms
	(2) Keywords	(2) Title and general terms	
	(3) General terms	(3) Keywords and general terms	

In algorithm, the step 1, define an array in which we assign all the metadata name (in our case names is: Title, Keywords and General Terms). In step 2, we have retrieved record against every metadata and stored in the list. In step 3, iterate the attribute array up to its length (in our case length is 3). In step 4, the combination function made all the possible combinations depending on the value of i, if the value i is 1 than the algorithm create all possible combination using one metadata parameter at a time and save it in a file using step 5 and 6 and then map them with their respective label, if the value is 2, then the algorithm create all possible combination using two metadata parameters etc.

### Preprocessing

Generally, some transactions in the datasets are incomplete: lacking attribute values (Missing Value), containing noisy data (meaningless data) etc. Tokenization is the first step of preprocessing. In this process, text can be divided into a set of meaningful pieces. These pieces are called tokens. In our scenario, we have divided the sentences into words. For this, we have used the Natural Language ToolKit (NLTK1), which is the best known and most used Natural language processing (NLP) library^34^. Limited number of records in dataset contain missing values, which is ignored. After tokenization, some of the punctuations are considered as tokens which is unnecessary (not meaningful). Therefore, we have removed all these unnecessary punctuations by using NLTK library. Stop words from all metadata parameters of datasets are removed using NLTK library. NLTK matches its own list of stop words with the tokenized list and then performed stop word removal from the corpus. Stemming is performed by using porter stemmer algorithm (Porter, 1980), which converts all the terms of a text into their root terms. The stemming algorithm is applied on all the metadata of both datasets.

### Vectorization

Most of the similarity measures and machine learning algorithms often take numeric vector as an input. Performing any operation on a text, document need to be converted into a numeric vector. Count based approaches and Semantic based approaches is used to convert text into numeric vector. Count based techniques in research articles classification approaches are: (1) One Hot Encoding (2) Bag of Word (BOW) or Term Frequency (TF), (3) Term Frequency and Inverse document frequency (TFIDF) and semantic based approaches are: (1) Glove (2) Fast Text and (3) Word2Vec.

**Figure 3:**
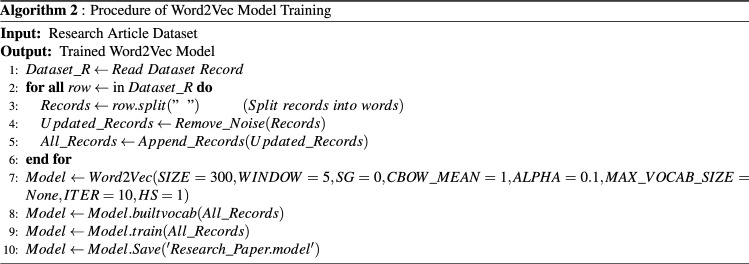
W2V model Training.

The current state-of-the-art approaches are^9–14^ for research article classification, employed conventional statistical measures like one hot Encoding, BOW, and TFIDF etc. Due to which they have not considered semantic and context due to which classification decision may affect. For considering this proposed work used Word2Vec model. For training Word2Vec model we have used both datasets instead of using already trained dataset by Google. For training we have used Algorithm 2 (presented in Fig. 3).

In step 1, all records of dataset are assigned to list. In step 2, iterate all the indices of the list. In step 3 and 4, sentences are split into words, then noise and stop words are removed. In step 5, all words are stored into another list. In step 7 we have defined a Word2Vec model is defined with all its parameter. These parameter values are not statically assign, we have found the optimum values after performing several round of experiments. Moreover, we have select those value on which result is maximum, by changing parameter values the results start decreasing. In step 8, Word2Vec model, first builds a vocabulary for training from the list which contains records of the dataset. In step 9, the model is trained on the dataset according to parameter which is already defined in step 7. In last step, model is saved in a model extension file for late use.

### Text conversion

**Figure 4:**
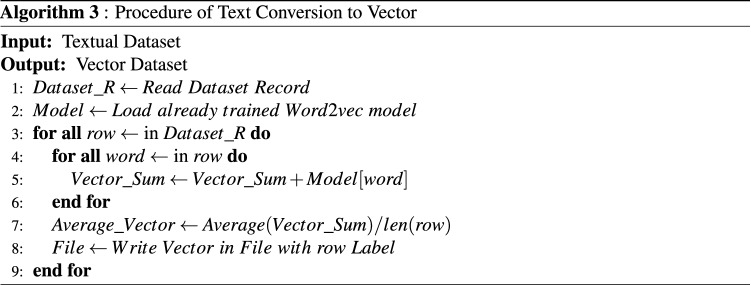
Conversion of text into vector.

The trained Word2Vec model has generated a vector of 75 * 4 lengths which consists of 300 elements. Each instance of record consists of random number of words, which is then combined to a single vector by considering context of all word vectors. The conversion of text into vector is performed by using Algorithm 3 (presented in Fig. 4).

In step 1 of this procedure, list is defined, and all the records of a dataset are assigned to list. In Step 2, trained Word2Vec model is loaded. In step 3, all the records of the dataset from the list are iterated. In step 4 iterate the individual record of words. In step 5, each word traverse from trained Word2Vec model. This model generates a vector of 75 * 4 length, which is added to previous word vectors if exists. In step 7, average of the individual record vectors is calculated and stored in list. In step 8, the generated vector is stored in a CSV file with their respective label.

### Similarity measure

Similarity of two documents corresponds to the correlation between the vectors. This is quantified as the cosine of the angle between two vectors. The standard formula for cosine similarity is given in Eq. 1 below:

1 $$\begin{aligned} CosineSimilarity (D_n ,D_m )=\frac{\sum _{i-1}^{n}D_{n_{i}},D_{m_{i}}}{\sqrt{\sum _{i-1}^{n}D_{n_{i}}^{2}}\sqrt{\sum _{i-1}^{n}D_{m_{i}}^{2}}} \end{aligned}$$

where $D_n ,D_m$ represent document 1 and document 2. So we have used cosine similarity for finding similarity between two documents.

### Single label classification (SLC)

**Figure 5:**
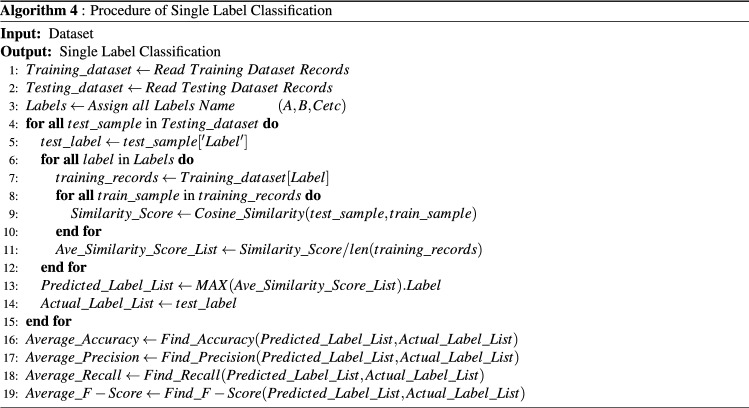
Procedure of single label classification.

The proposed approach is evaluated on both datasets for single-label document classification. In case of single label classification, test document is given to the system as an input, the system extract the metadata features from the test document. Thereafter, system transform these textual feature into numerical form by using semantic based train Word2Vec model. Afterwards, the system calculates the similarity score of a test document with every individual category papers. The system has calculated an average of calculated score of a test document with the score of individual category papers. The average score represents the individual category similarity score. At the end system has select the category which have highest average similarity score. The Algorithm.4 (presented in Fig. 5 ) used for the single label classification procedure.

In SLC procedure, in step 1 and step 2, training and testing dataset and saved in the list. Step 3 defined the list for label names. From step 4 to 7 labels are extracted form testing and training data. In step 8 and 9, similarity of each test sample is calculated with training samples. In step 11, average similarity score is calculated for each category of papers. The Eq. 2 used for finding the Average similarity score of a category:

2 $$\begin{aligned} AS_{c}=\frac{1}{n} \sum _{i=1}^{n}SS_{^{C_{i}}}({T_{p},P_{C_{i}}}) \end{aligned}$$

whereas $AS_{c}$ presents the average similarity score of the individual category (A, B, C. . . . . . K), $T_{p}$ represent test paper, $P_{C_{i}}$ represents individual category papers.

In step 13, we have picked the highest average similarity score label is considered as a predicted category of a test sample. The Eq. 3 used for selecting the predicted category.

3 $$\begin{aligned} Predicted Category=Max(AS_{a},AS_{b},AS_{c} \ldots AS_{k}) \end{aligned}$$

From step 16 to 19, accuracy, F1-score, precision and recall are calculated (Equation are given in “Evaluation parameters” Section).

### Multi-label classification (MLC)

**Figure 6:**
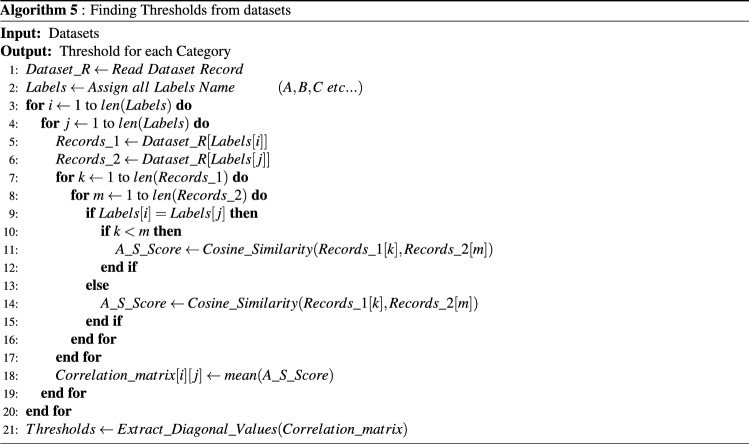
Finding threshold algorithm.

In case of multi label classification, for assigning multi label to the documents you have required some threshold value. In existing state-of-the-art, researchers have picked the method of selecting threshold value either asking from domain expert or by choosing some arbitrary values and then ensuring them on the basis of trial and error on dataset, which is a time consuming task. We have argued that dependence on domain experts or on some arbitrary value does not adequately serve the said purpose. Our proposed work focused on designing a scheme which can help in finding threshold values based on rigorous analysis of the dataset. For this we have find the correlation matrix between the categories of a research articles. Each value in a correlation matrix is the average similarities score between two categories of research articles. The algorithm 5 (presented in Fig. 6 ) is used to find threshold values for each category:

Step 1 load the dataset records in the list. Step 2 defined a list for label names. Step 3 to 6 read labels from Labels list and extract records against those labels. From step 7 to step 18, the algorithm finds the average similarity score between extracted records of both labels and saved in a correlation matrix on its specific index. The correlation matrix of a dataset is shown in Eq. 4:

4 $$\begin{aligned} Correlation Matrix (D_{n})= \begin{bmatrix} SS_{C_{1}C_{1}} &{} SS_{C_{1}C_{2}} &{}\ldots&{}SS_{C_{1}C_{m}} \\ .&{} &{} \\ .&{} &{} \\ SS_{C_{n}C_{1}} &{} SS_{C_{n}C_{2}} &{}\ldots&{}SS_{C_{n}C_{m}} \end{bmatrix} \end{aligned}$$

The specific value of a correlation matrix is defining by the Eq. 5:

5 $$\begin{aligned} SS_{C_{n}C_{m}}=\frac{1}{M*N}\sum _{i=1}^{M}\sum _{j=1}^{N}SS(P_{C_{n_{i}}},P_{C_{m_{j}}}) \end{aligned}$$

In step 21, diagonal values of a correlation matrix (shown in Eq. 4) are extracted which represent the threshold value for different labels which are assign in Labels list (Eq. 6).

6 $$\begin{aligned} M_{T}(D_{n})=(SS_{C_{1}C_{1}},SS_{C_{2}C_{2}},SS_{C_{3}C_{3}}\ldots SS_{C_{n}C_{m}}) \end{aligned}$$

For experimental purpose, before performing multi-label classification we have found a threshold values by using the above Algorithm 5 for all possible metadata combination of both dataset (ACM & JUCS). From both datasets, we have selected the multi label instances of (H, I, D, F and K) categories and (H, D and I) categories from JUCS and ACM datasets respectively. The reasons of choosing these categories of both datasets is that these categories cover maximum amount of record and another major reason is that we intend to compare our outcomes to one similar state-of-the-art study which has picked these categories. Moreover, we have calculated the average threshold values using Training datasets. The average threshold values of different combination of a JUCS and ACM datasets are illustrated in the Tables 3 and 4 respectively:

**Table 3:** JUCS dataset.

Datasets	Combination	D	F	H	I	K
JUCS	Title	0.36	0.35	0.42	0.32	0.34
	Keywords	0.42	0.37	0.43	0.38	0.42
	Title and keyword	0.39	0.42	0.42	0.41	0.47

**Table 4:** ACM dataset.

Datasets	Combination	D	H	I
ACM	Title	0.14	0.10	0.11
	Keywords	0.15	0.14	0.12
	General terms	0.44	0.50	0.52
	Title and keyword	0.21	0.22	0.15
	Title and general terms	0.27	0.32	0.25
	Keyword and general terms	0.26	0.31	0.24
	Title, keyword and general terms	0.32	0.22	0.32

**Figure 7:**
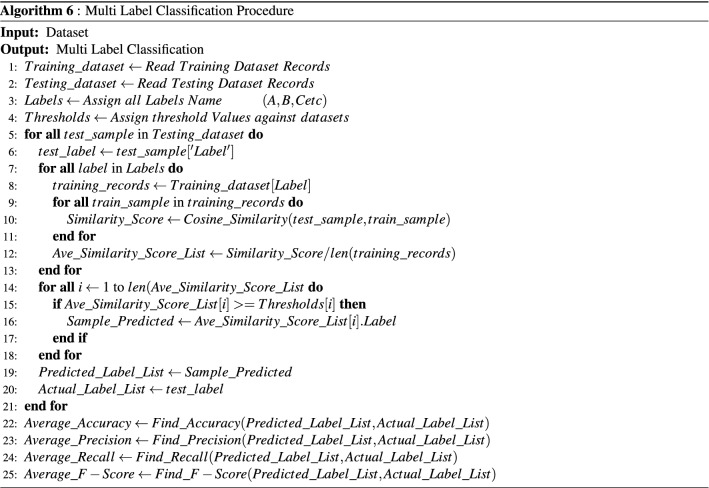
Multi label classification procedure.

After finding threshold values, we have just compared the average similarity score of a test paper with every individual category with their respective threshold value. If the category score satisfies the threshold value, these categories is selected as a final list of predicted categories. The multi-label classification performed by using Algorithm 6 ( presented in Fig. 7).

In steps 1,2 and 3, training and testing labels are retrieved from their respective files. In step 4, thresholds are calculated with the help of Algorithm 5 (presented in Fig. 6). From step 5 to 21, labels are predicted for each test sample. In step 5, labels of each test sample is retrieved. A simple may have multiple actual labels. From step 7 and 13, average similarity score between test sample and all training records are calculated. In step 8, all record for the single label are extracted from training dataset. In step 9 to 11, similarity score between selected test sample and each record of the extracted training records are calculated. In step 12, average similarity score is calculated for selected label. In step 14 to 18, labels are predicted for each training record. In step 15, if average similarity score of a label is greater or equal to threshold value, then assign that label to the sample predicted for that record. In this way, maybe multiple labels satisfy the condition, in that case multiple label will be assigned as predicted label to the record. In step 19, predicted and actual labels for each testing record are stored in their respective lists. In step 22 to 25, accuracy, precision, recall and F1-score are calculated for predicted and actual labels (Equation are present in “Evaluation parameters” section). The multi-label classification results are mentioned in result section.

### Experimental setup

Before performing experiments we have employed the stratified k fold cross validation on datasets. This cross validation is a variation of simple KFold that return stratified folds. These folds are made by preserving the percentage of sample for each class or category. Moreover, we have used the value of K is 5, because we have conducted some experiment and from experiment we have concluded that by increasing the value of K than you got a small proportion of testing dataset while by decreasing the value of K you have move towards the over fitting situation. So by using stratified k fold cross validation we have divided our each dataset into 5 folds, in which each fold contain equal amount of samples from each category. At a time from these 5 folds, one fold act as a testing dataset and the remaining four fold act as training datasets. So for each dataset we have repeated experiment 5 time and reported the results by taking the average of these 5 experiments.

### Evaluation parameters

To evaluate the results of our proposed technique, the standard formula of Precision, Recall and F-measure is calculated. The main reason behind the selection of these evaluation parameters is the frequent reporting of these parameters in literature. The formula of these measures is somehow changed for single label and multi-label classification, because in multi-label classification the partially correct concept is used in these formulas.

#### Single label classification parameters

The proposed approach have evaluated on both datasets for single-label classification. The evaluation parameter used for single label classification are given below:

7 $$\begin{aligned} AverageAccuracy= & {} \frac{1}{f}\sum _{f=1}^{f=5}\frac{1}{c}\sum _{i=1}^{i=c}\left(\frac{True Positive (C_{i})+True Negative (C_{i})}{True Positive (C_{i})+TrueNegative (C_{i})+False Positive (C_{i})+FalseNegative (C_{i})}\right) \end{aligned}$$

8 $$\begin{aligned} Average Precsion= & {} \frac{1}{f}\sum _{f=1}^{f=5}\frac{1}{c}\sum _{i=1}^{i=c}\left(\frac{True Positive (C_{i})}{True Positive (C_{i})+False Positive (C_{i})}\right) \end{aligned}$$

9 $$\begin{aligned} Average Recall= & {} \frac{1}{f}\sum _{f=1}^{f=5}\frac{1}{c}\sum _{i=1}^{i=c}\left(\frac{True Positive (C_{i})}{True Positive (C_{i})+False Negative (C_{i})}\right) \end{aligned}$$

10 $$\begin{aligned} Average F-Score= & {} \frac{1}{f}\sum _{f=1}^{f=5}\frac{1}{c}\sum _{i=1}^{i=c}\frac{2(Precsion(C_{i})*Recall(C_{i})))}{(Precsion(C_{i})+Recall(C_{i}))} \end{aligned}$$

In above equations small c represent number of categories, capital $C_{i}$ represent the individual Category and f represent fold of stratified k fold cross validation.

#### Multi label classification parameters

The proposed approach have evaluated on both datasets for multi-label classification. The following evaluation parameters for multi-label document classification proposed by Godbole and Sarawagi^35^. These formulas are described below:

11 $$\begin{aligned} Average Accuracy= & {} \frac{1}{f}\sum _{f=1}^{f=5}\frac{1}{n}\sum _{i=1}^{n}\frac{\left| Predicted Labels \bigcap Actual Labels\right| }{\left| Predicted Labels \bigcup Actual Labels\right| } \end{aligned}$$

12 $$\begin{aligned} Average Precsion= & {} \frac{1}{f}\sum _{f=1}^{f=5}\frac{1}{n}\sum _{i=1}^{n}\frac{\left| Predicted Labels \bigcap Actual Labels\right| }{\left| Predicted Labels\right| } \end{aligned}$$

13 $$\begin{aligned} Average Recall= & {} \frac{1}{f}\sum _{f=1}^{f=5}\frac{1}{n}\sum _{i=1}^{n}\frac{\left| Predicted Labels \bigcap Actual Labels\right| }{\left| Actual Labels\right| } \end{aligned}$$

14 $$\begin{aligned} Average F-Score= & {} \frac{1}{f}\sum _{f=1}^{f=5}\frac{1}{n}\sum _{i=1}^{n}\frac{2(Precsion)(Recall)}{Precsion+Recall} \end{aligned}$$

## Results

In this section we present the details about the results that have been obtained by applying the proposed methodology. We have evaluated our datasets for single as well as multi-label classifications. In both classifications we have conducted experiment on individual as well as on combination of metadata features. Results presented in following sections.

### Single label classification

For single label Classification, the algorithm 4 (presented in Fig. 5), predict only that category which have highest average similarity score with test paper. For the evaluation of our proposed techniques, single label instances of (H, I, D, F and K) categories and (H, D and I) categories are collected from JUCS and ACM datasets, respectively. The reasons of choosing these categories of both datasets is that these categories cover maximum amount of record as compare to other categories. To analyze the contribution of metadata, several experiments were performed for both individual and combined effect of metadata.

#### Single metadata parameters

**Figure 8:**
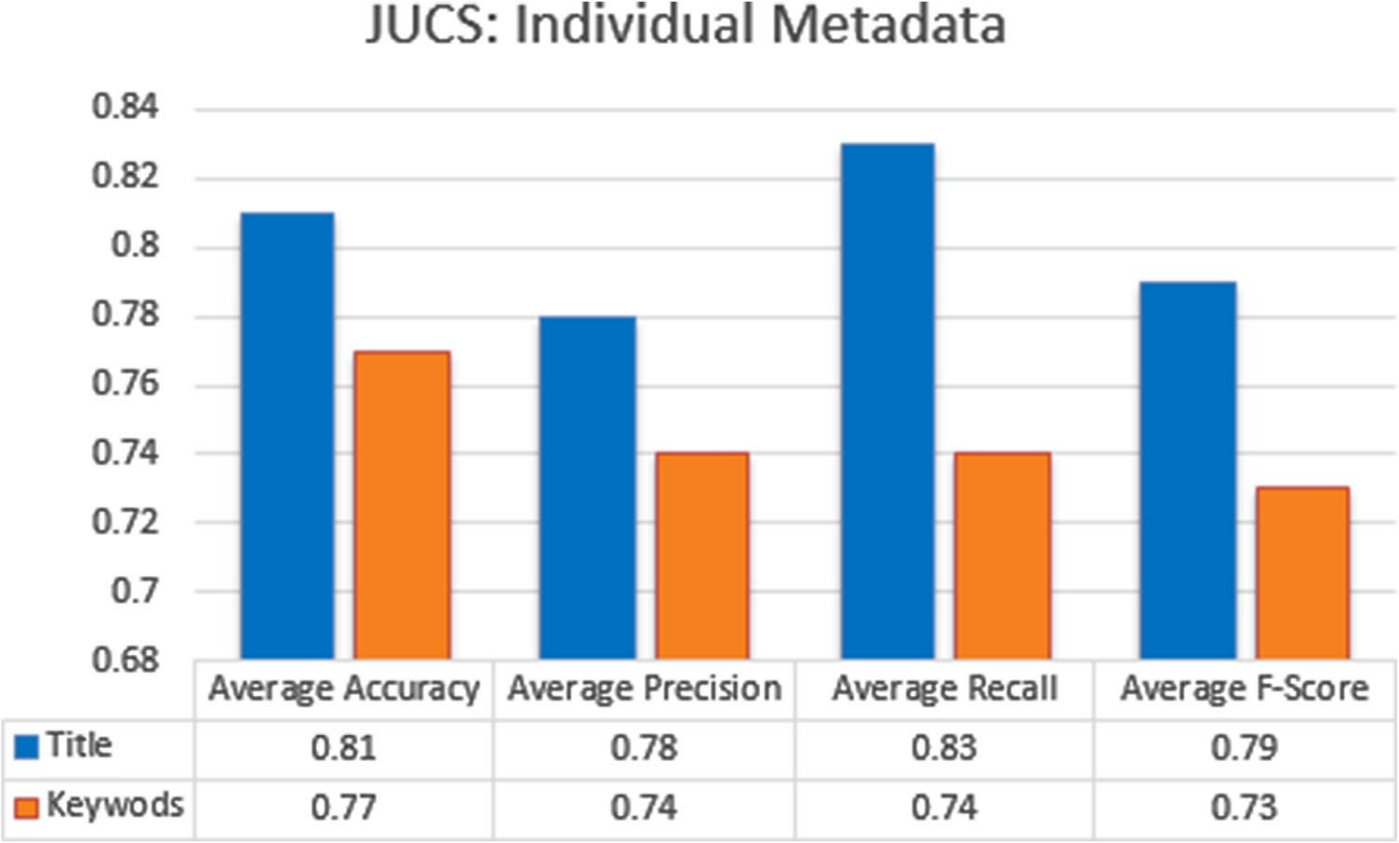
JUCS (individual metadata).

**Figure 9:**
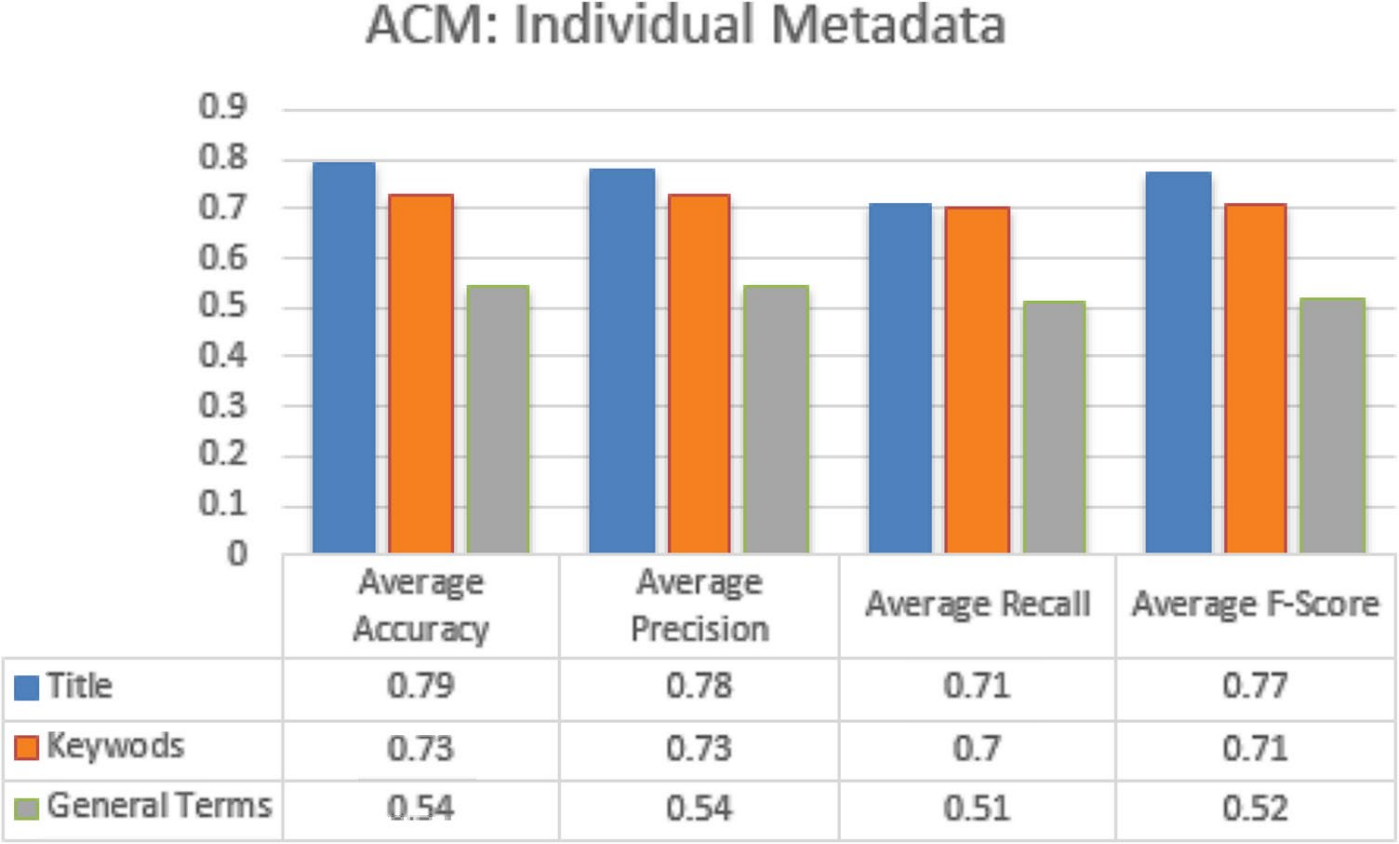
ACM (individual metadata).

The classification based on individual metadata features contributed more in achieving good results. For every individual metadata feature, accuracy, precision, recall and f-measure score was calculated for all the categories, and average accuracy, precision, recall and f-measure score is obtained by calculating the average of all the categories. In case of JUCS datasets, title metadata achieved the highest average Accuracy 0.81, Precision of 0.78, Recall of 0.83 and F-measure of 0.79, then Keywords metadata as shown in the Fig. 8. In Case of ACM datasets, the similar results are achieved in case of title, as title metadata in ACM outperformed other metadata with average Accuracy 0.79, Precision of 0.78, Recall of 0.71, and F1-measure 0.77, followed by Keywords and General Terms parameter as shown in the Fig. 9. Similar behavior of title metadata in both datasets shows, that title hold a strong potential in case of single label classification. As the title represent main idea of a research work so it contains such like words which specifically denote the particular subject. However, the articles belong to same category are more similar as compare to different categories articles, that’s why, when a test document is given to the system as an input, their exist more chances that its similarity score will be high with their actual paper categories as compared to other categories.

#### Double metadata parameters

**Figure 10:**
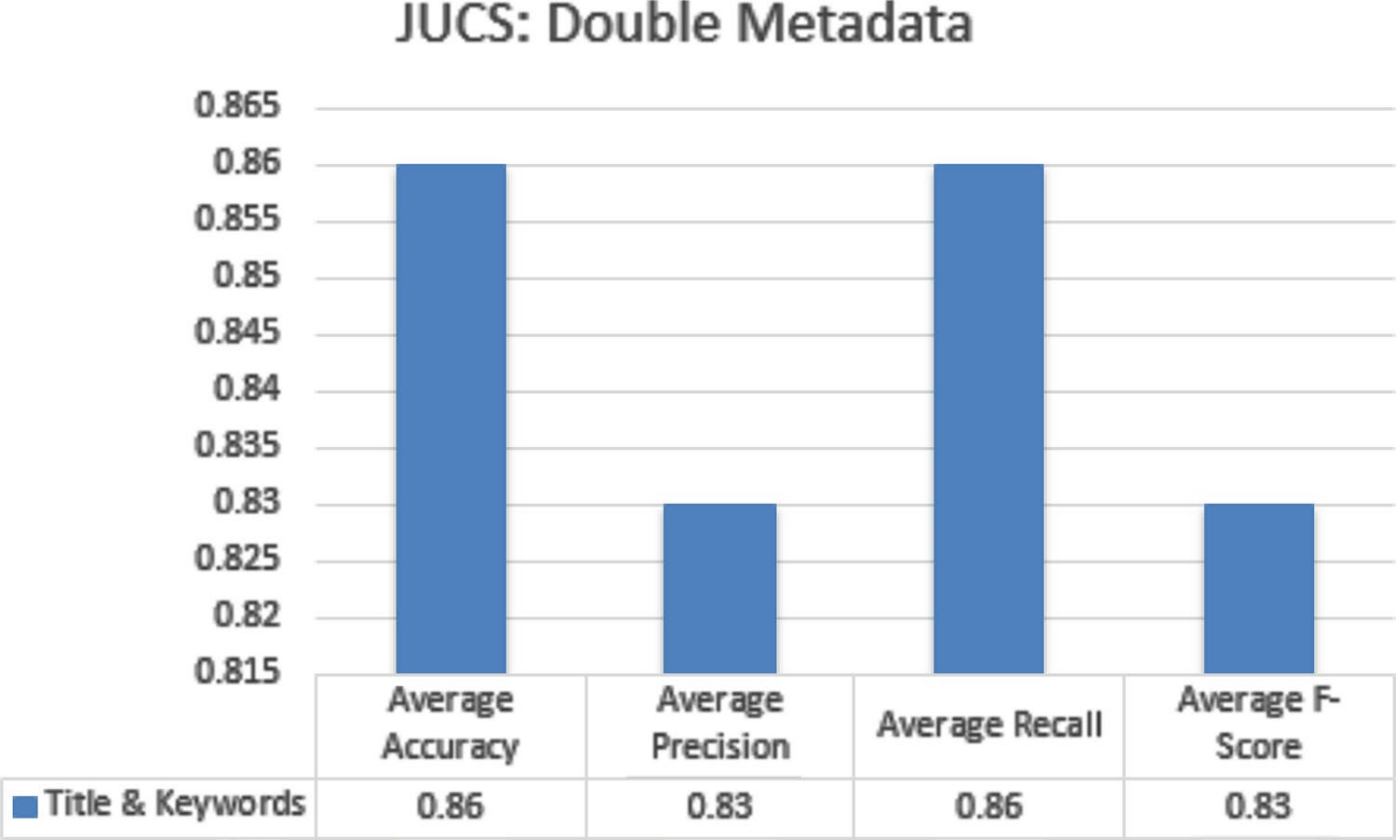
JUCS (double metadata).

**Figure 11:**
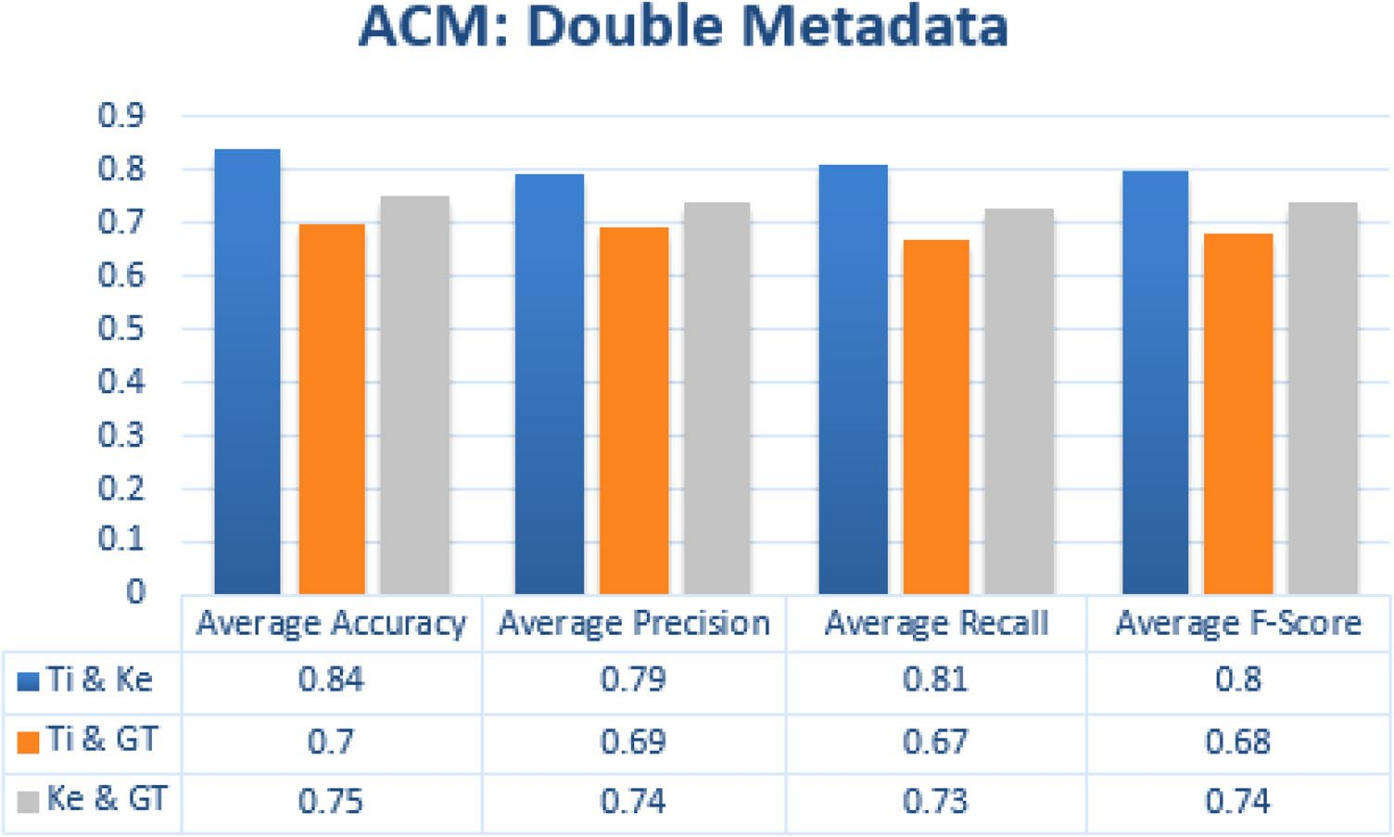
ACM (double metadata).

In double metadata parameter every possible combination of two metadata parameters is exploited to obtain average accuracy, precision, recall and f-measures scores. In case of JUCS dataset there is only one combination of two metadata features “Title + Keywords” which obtained average Accuracy of 0.86, average Precision of 0.83, average Recall of 0.86 and average F1-measure is 0.83 which is shown in the Fig. 10. In case of ACM datasets there are three double metadata parameter combinations “Title + Keywords”, “Title + General Terms” and “Keywords + General Terms”. The “Title + Keywords” combination outperformed other combination with the average Accuracy of 0.84, average precision of 0.79, average Recall of 0.81 and average F1-measure 0.8. The second top scored combination is “Title + General Terms” and the third one is “Keywords + General Terms” shown in the Fig. 11. In case of Bi metadata features combination, while adding the keywords metadata with title metadata can improved the results of single label classification of research articles. The basic reason of improvement of classification is that, while adding keywords metadata it provide some more specific words which represent the subject of the paper. These words combine with Title words and classify the research article more accurately as compare to individual title words. The abbreviation of metadata parameters presented in the Figs. 10, 11 are (1) Ti: Title, (2) Ke: Keywords (3) GT: General Terms.

#### Triple metadata parameters

**Figure 12:**
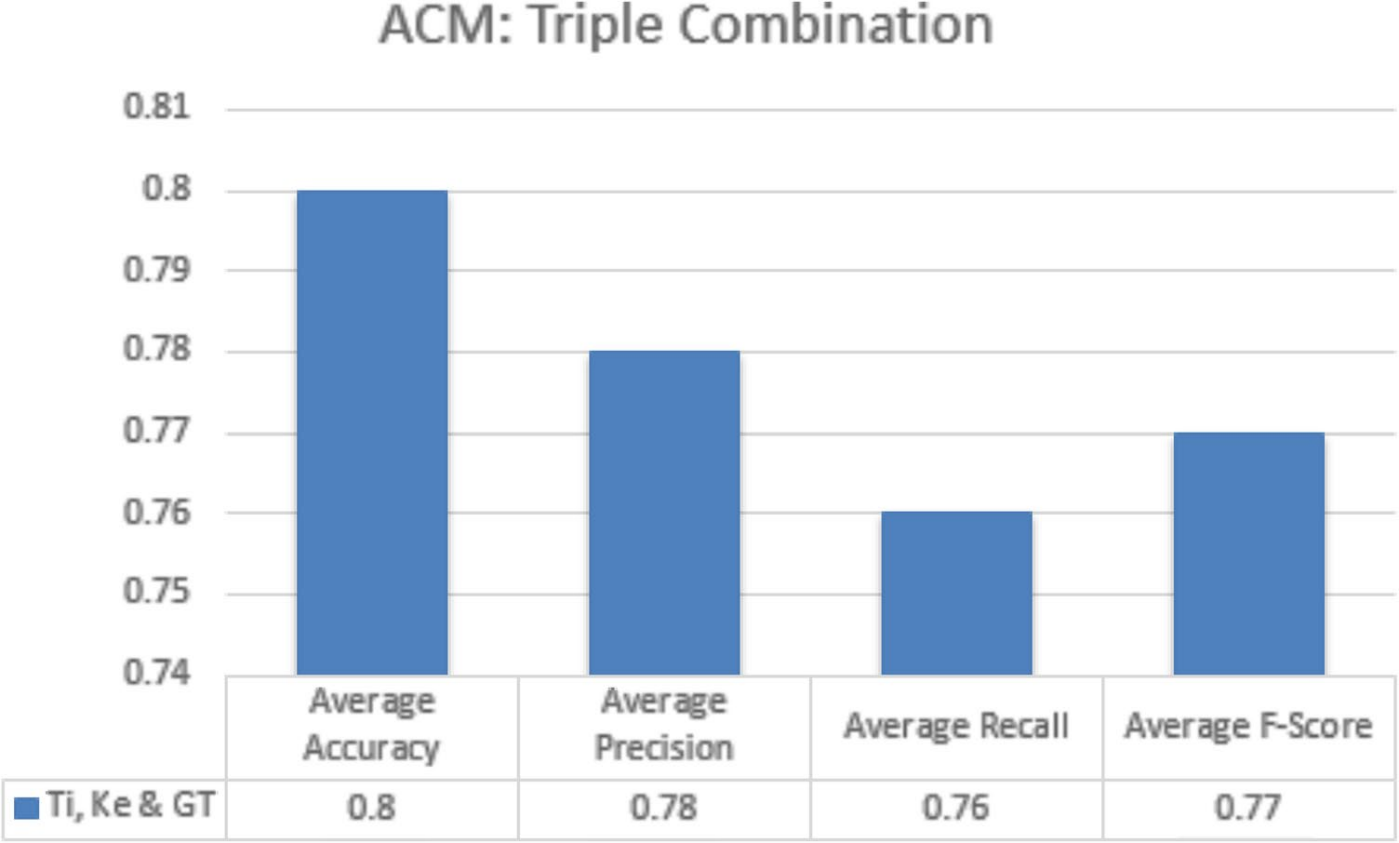
ACM (triple metadata).

In triple metadata parameter every possible combination of three metadata parameters is exploited to obtain average accuracy, precision, recall and f-measures scores. In case of JUCS dataset, there is no triple metadata combination while in case ACM dataset there is only one triple metadata combination which was “Title + Keywords + General Terms”. The results obtained by this combination was lower as compare to “Title + Keywords” combination. Similarity of general term records are high as compared to title and keywords in different categories. Addition of general term with title and keywords negatively affected the classification results, due to decrease in diversification of records in the dataset. The results obtained by this combination are given in Fig. 12. The abbreviation of metadata parameters presented in the Fig. 12 (1) Ti: Title, (2) Ke: Keywords (3) GT: General Terms

### Multi-label classification

Before evaluating our proposed approach for performing multi-label classification, in first step, threshold values is calculated with the help of algorithm 5 (presented in Fig. 6) for all possible metadata combinations of both datasets(Threshld values presented in Tables 3 and 4.

After defining all threshold value, now we have performed multi-label classification by using multi-label classification algorithm. The algorithm finds average similarity score of a test document with every individual category papers. These average similarity score of each category was compared with their respective threshold. The category score which have met their threshold value is selected as a predicted category. For experiments, the multi- label instances of (H, I, D, F and K) categories and (H, D and I) categories from JUCS and ACM datasets respectively. The reasons of choosing these categories of both datasets is that these categories cover maximum amount of record and another major reason is that we intend to compare our outcomes to one similar state-of-the-art study which has picked these categories. Since comparison results are justified when major factors among the studies have been contemplated on the basis of same grounds. Similar to single label classification, we have analyzed the contribution of each metadata individually and collectively.

#### Single metadata parameters

**Figure 13:**
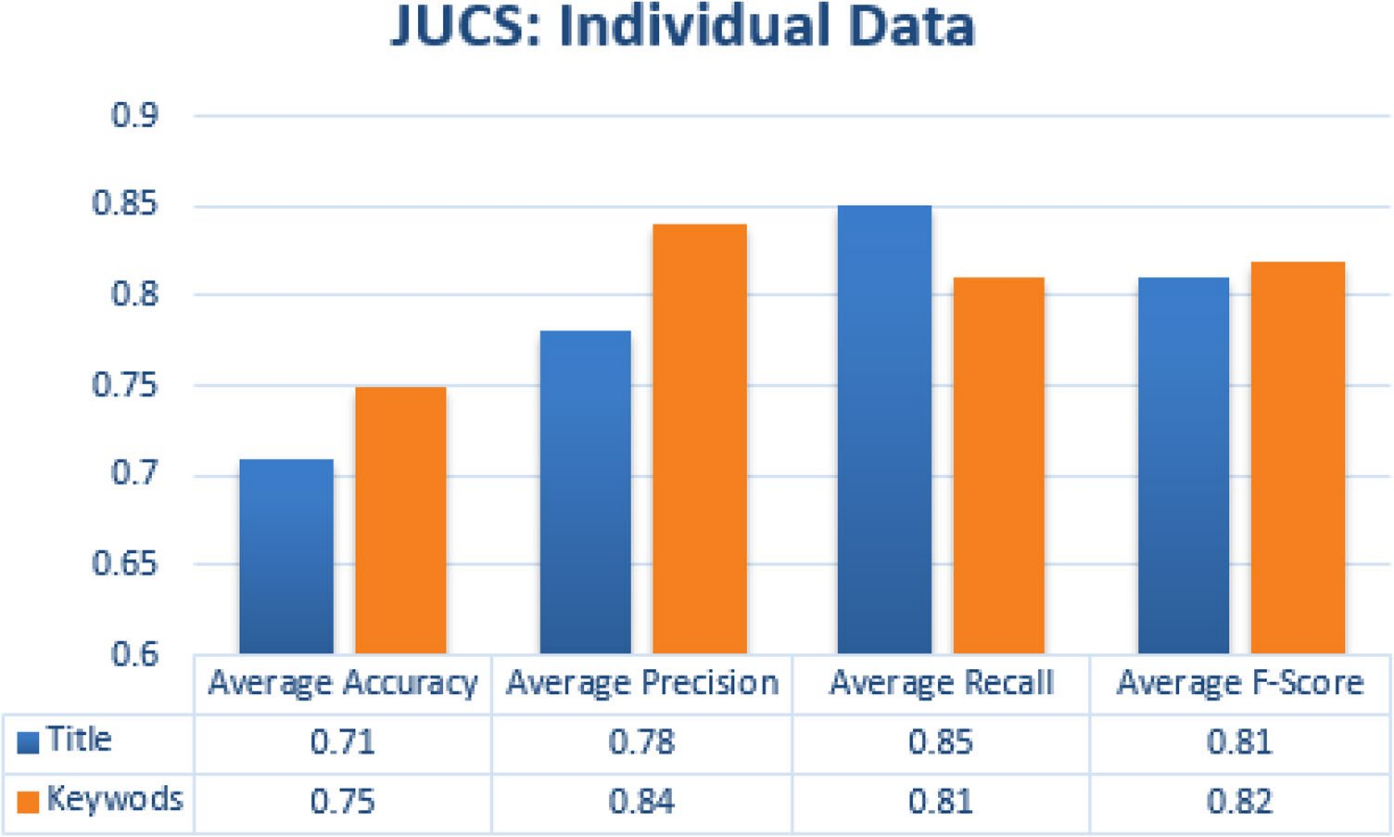
JUCS (individual metadata).

**Figure 14:**
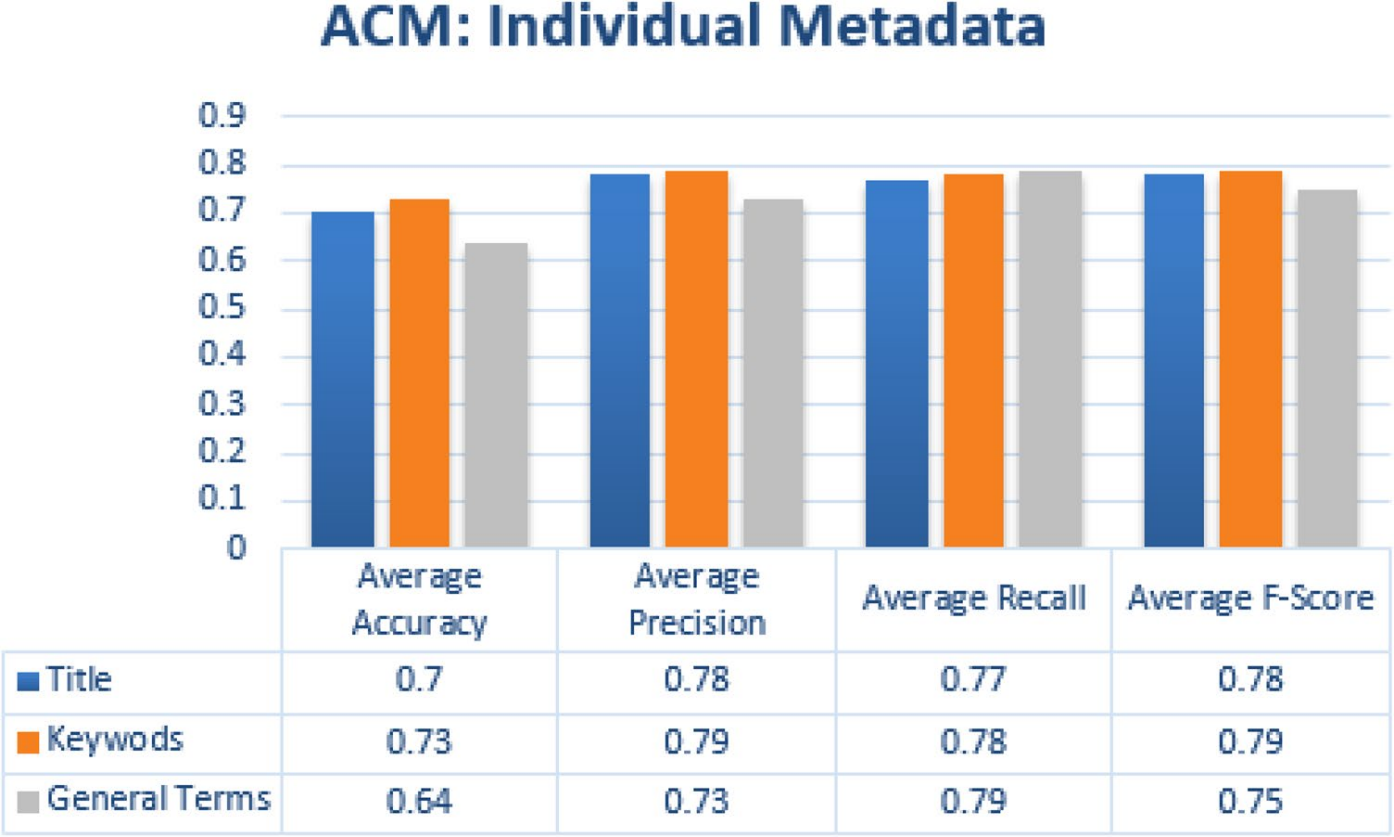
ACM (individual metadata).

Similar to single label classification we have also evaluated individual metadata features which helps us in finding some metadata features who’s individually contributed more in achieving good results. For every individual metadata feature average accuracy, precision, recall and f-measure score was obtained by calculating the average of all the categories. In case of JUCS datasets, Keywords metadata achieved the highest average Accuracy of 0.75 as shown in the Fig. 13. In Case of ACM datasets, the similar results were achieved in case of Keywords metadata, as Keywords metadata in ACM outperformed title and general terms with average Accuracy of 0.73 as shown in the Fig. 14. Above result shows that Keywords metadata in both datasets represent a strong potential in case of MLC. In SLC the title metadata is better than Keywords while in MLC Keywords is better than title. The reason of effectiveness of keywords in MLC is that, keywords contains words which represent different domains. However, these words are helpful in MLC as compare to title metadata which is better for SLC.

#### Double metadata parameters

**Figure 15:**
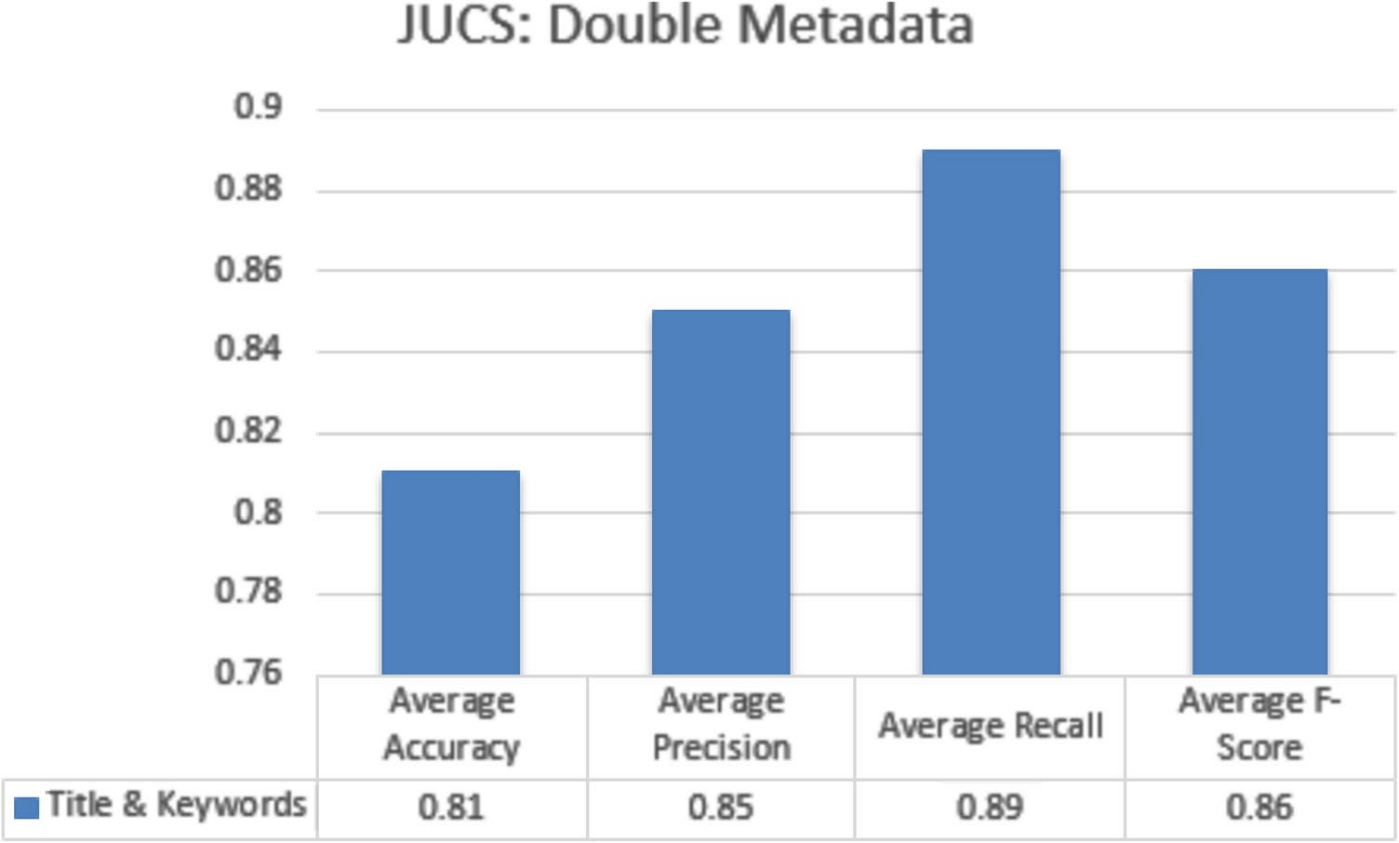
JUCS (double meta data).

**Figure 16:**
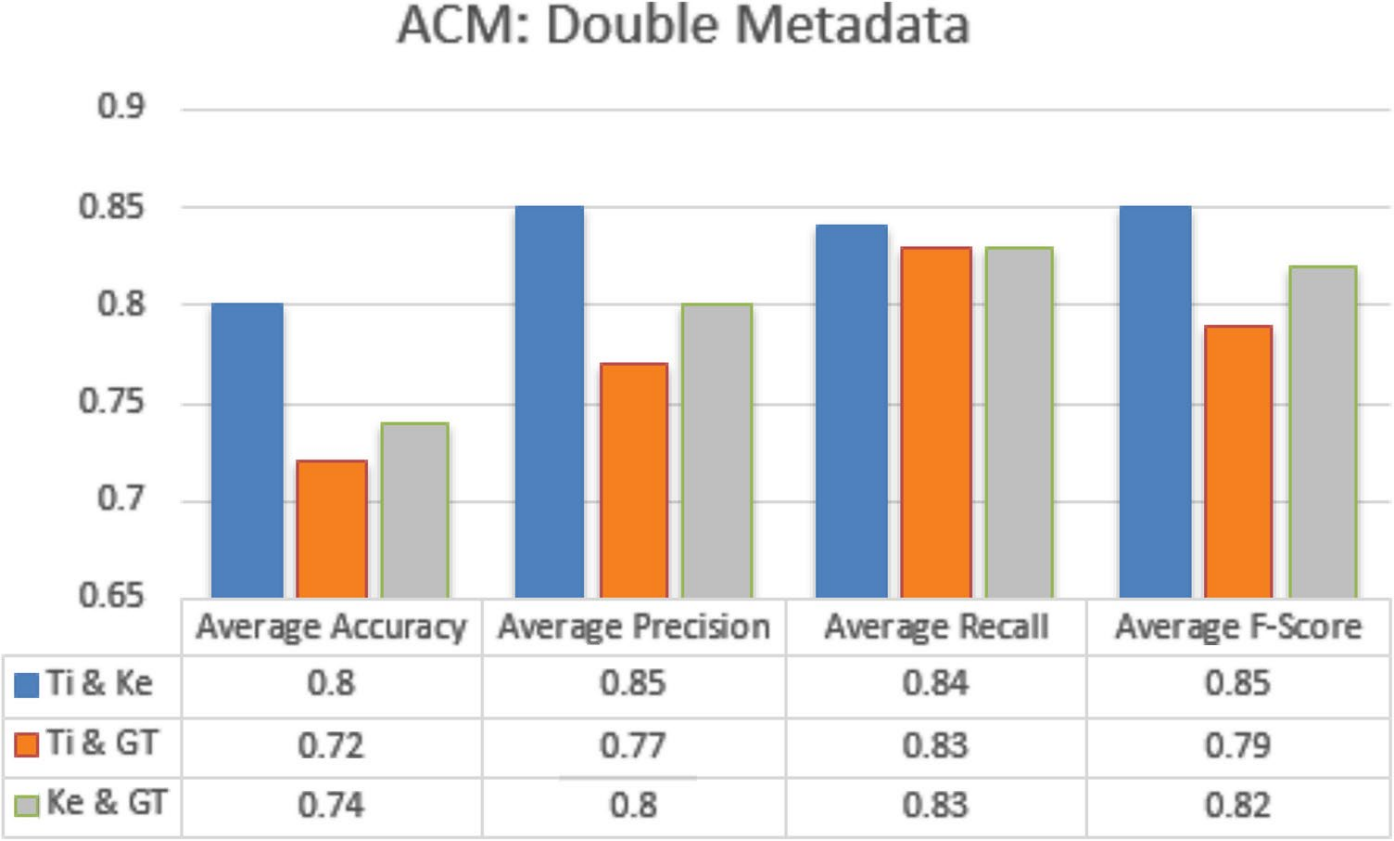
ACM (double meta data).

In double metadata parameter every possible combination of two metadata parameters are exploited to obtain average accuracy, Precision, Recall and F-measures scores. In case of JUCS dataset there is only one combination of two metadata features “Title + Keywords” which obtained average Accuracy of 0.81 shown in Fig. 15. In case of ACM datasets there are three double metadata parameter combinations “Title + Keywords”, “Title + General Terms” and “Keywords + General Terms”. The “Title + Keywords” combination outperformed other combination with the average accuracy of 0.80 shown in the Fig. 16. In case of Bi metadata features combination, title metadata and keywords metadata improve the results of MLC of research articles. Sometimes the keywords metadata contains words, which are generic in nature which mostly occur in different categories articles so it distracts classification algorithm to classify the research article. In these scenarios, by adding title with keywords metadata at least one of the subjects would be correctly classified. So that’s why the accuracy of multi-label classification has been increased by adding title with keywords metadata.

#### Triple metadata parameters

**Figure 17:**
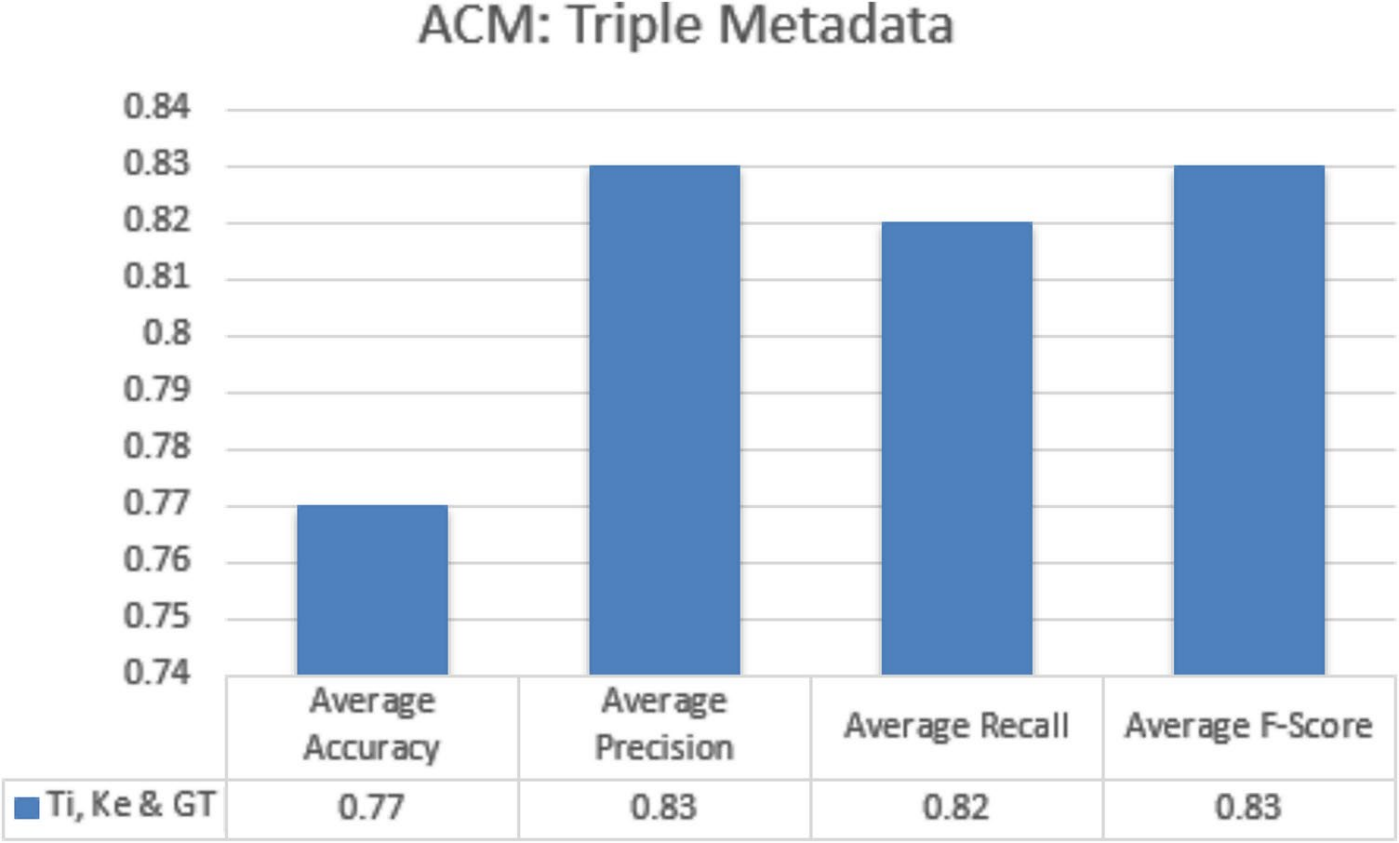
ACM (triple metadata).

In triple metadata parameter every possible combination of three metadata parameters are exploited to obtain average accuracy, precision, Recall and F-measures scores. In case of JUCS dataset, there is no triple metadata combination while in case ACM dataset there is only one triple metadata combination which is “Title + Keywords + General Terms”. Like SLC, in MLC the results obtained by this combination is lower as compare to simple “Title + Keywords” combination. Addition of general term with title and keywords negatively affect the classification results, due to decrease in diversification of records in the dataset. The results obtained by this combination are given in Fig. 17. The abbreviation of metadata parameters presented in the Fig. 11 are (1) Ti: Title, (2) Ke: Keywords (3) GT: General Terms.

### Comparison

**Figure 18:**
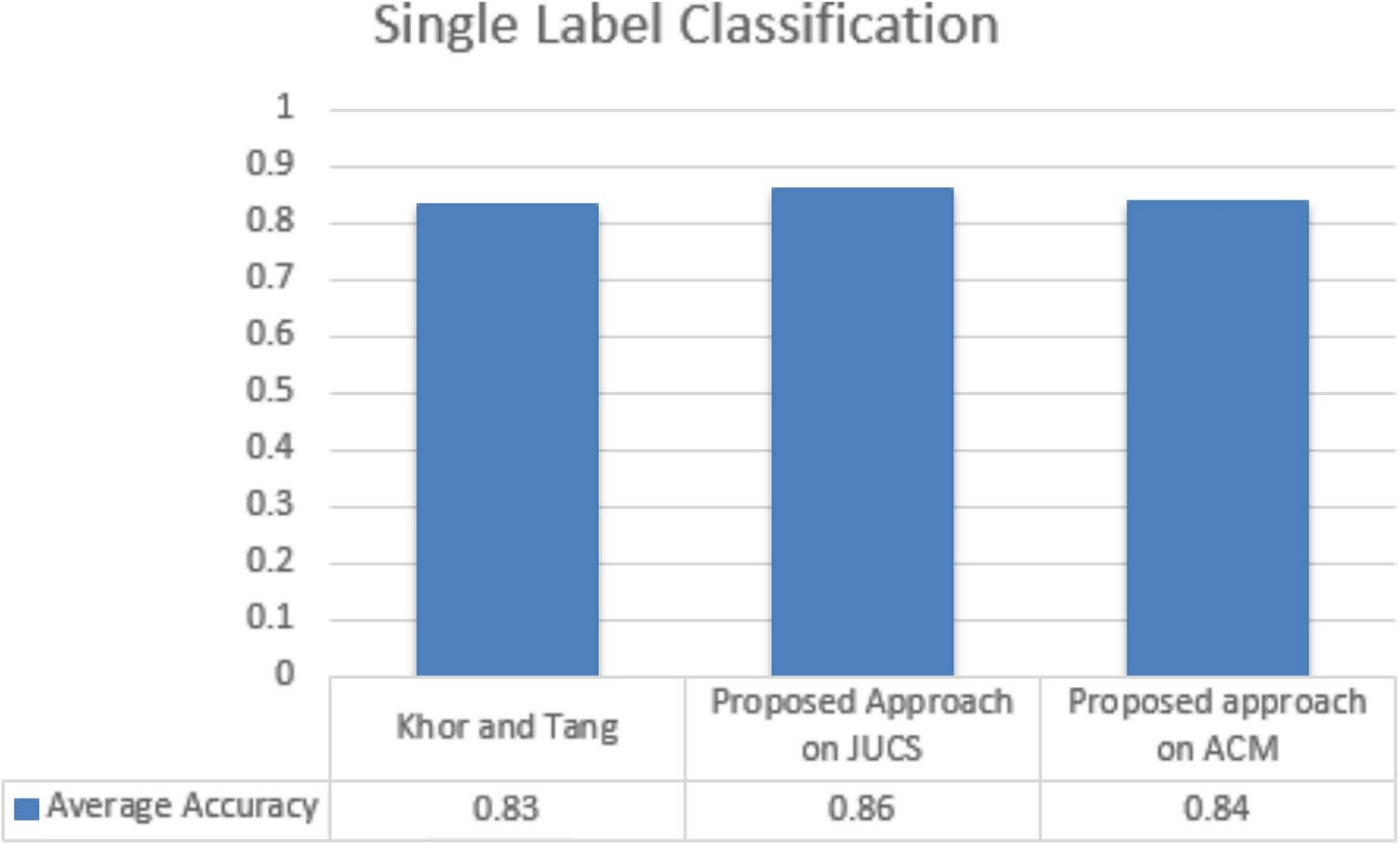
Single label classification comparison.

**Figure 19:**
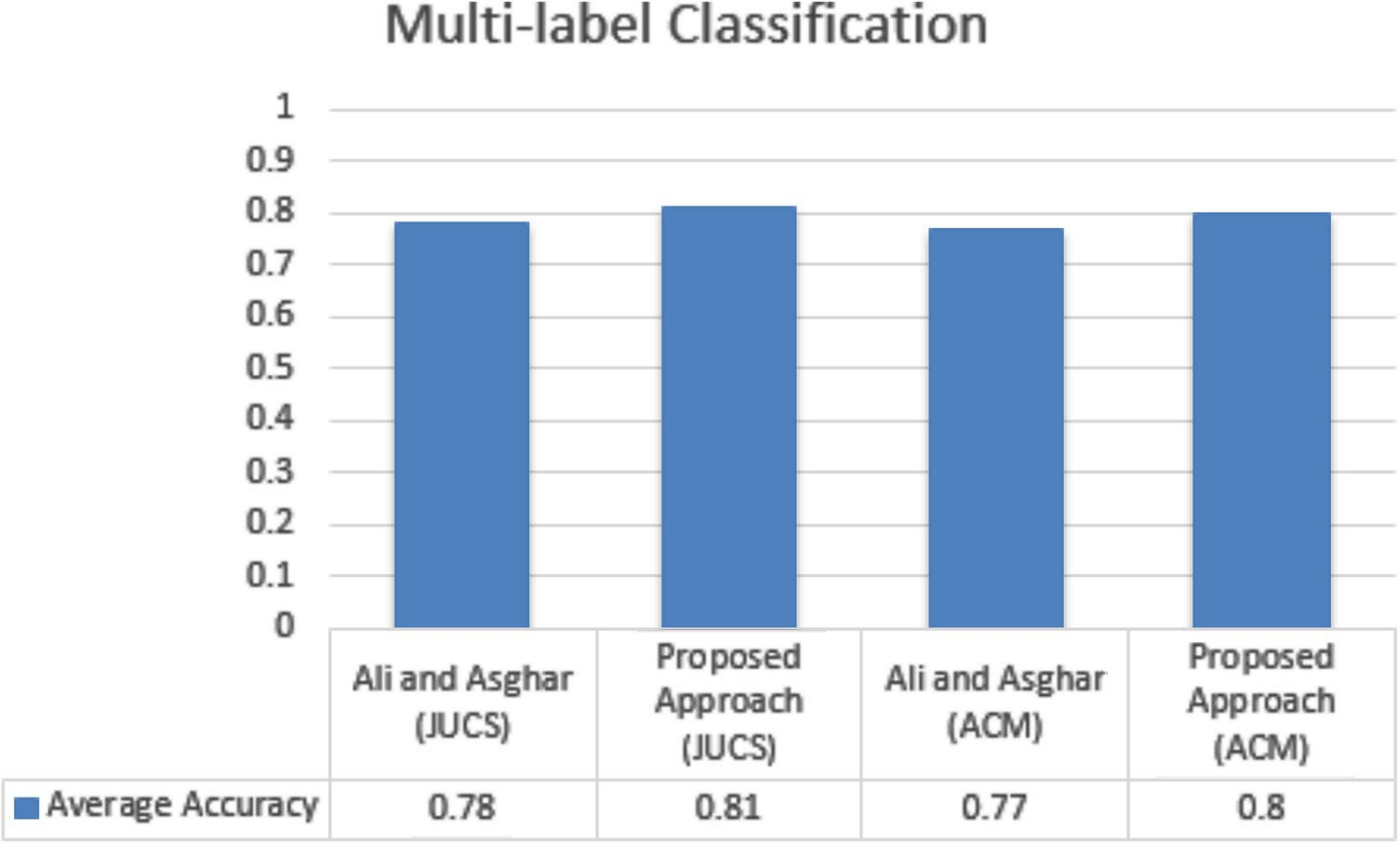
Multi-label classification results.

The document classification community has proposed multiple approaches for performing SLC and MLC. Most of these approaches have utilized the overall content of the research articles while some have prefer to harness metadata parameters due to unavailability of content. In this paper we have also utilized the freely available metadata (1) Title, (2) Keywords and (3) General Terms, for performing SLC as well as MLC. In case of SLC, proposed approach is compared with Khor and Tang^31^ which also utilizes the metadata of the research articles. For evaluation Khor and Tang collect 400 educational conference’s papers and performed SLC onto four topics such as “Intelligent Tutoring System”, “Cognition”, “E-Learning” and “Teacher Education”. This approach has used different classifier for classification and achieved average accuracy up to 0.83. However, this approach does not provide their dataset and in-depth detail of their methodology. The comparison results are shown in the Fig. 18. The approach proposed by Khor and Tang considered a very few numbers of papers for the SLC. However, our datasets contain more than fifty thousand research articles. In case of MLC, proposed work is compared with the results of approach proposed by Ali and asghar in 2018. Their approach utilized metadata of research articles of both JUCS and ACM datasets. The comparison results are shown in the Fig. 19. From the Fig. 19 it is conclude that both datasets, our proposed technique achieved good results as compare to Ali and Asghar^32^ for performing Multi-label Classification.

The table 5 presented the overall results of our experiments. From the table we have observed that, in case of SLC our proposed approach have achieved average accuracy of 0.84 and 0.86 on ACM and JUCS dataset respectively by using title & keywords Combination while the Khor and Ting approach have achieved average accuracy of 0.83 by using Keywords as a feature, while in case of MLC our proposed approach have achieved 0.80 and 0.82 average accuracy on ACM and JUCS dataset respectively by using title & keywords Combination, while the Ali et al have achieved average accuracy of 0.77 and 0.78 on ACM and JUCS dataset respectively by Title & Keywords Combination.

**Table 5:** Overall results.

Approaches	Datasets	Classification type	Combinations	Features	Average accuracy
Proposed Approach	ACM Dataset	Single Label Classification	Individual metadata	Title	0.79
				Keywords	0.73
				General Terms	0.54
			Double metadata	Title Keywords	0.84
				Title & Generals Terms	0.70
				Keywords & Generals Terms	0.75
			Triple metadata	Title, Keywords & Generals Terms	0.8
		Multi Label Classification	Individual metadata	Title	0.7
				Keywords	0.73
				General Terms	0.64
			Double metadata	Title Keywords	0.80
				Title & Generals Terms	0.72
				Keywords & Generals Terms	0.74
			Triple metadata	Title, Keywords & Generals Terms	0.77
	JUCS Dataset	Single Label Classification	Individual metadata	Title	0.81
				Keywords	0.77
			Double metadata	Title Keywords	0.86
		Multi Label Classification	Individual metadata	Title	0.71
				Keywords	0.75
			Double metadata	Title Keywords	0.81
Khor and King et al	400 Article	Single Label Classification	Individual metadata	Keywords	0.83
Ali and Asghar et al	ACM	Multi Label Classification	Double metadata	Title Keywords	0.77
	JUCS				0.78

## Discussion

We performed document classification based on SLC and MLC. The proposed approaches have been classified into two groups in the literature: (1) content-based approaches and (2) metadata-based approaches. Because of the variety of attributes, most of these approaches used content-based parameters. However, the content of the papers is not readily available, limiting the breadth of content-based techniques. Due to the limited number of features, very few researchers have used openly available information to categorize the paper, and as a result, these approaches have failed to provide promising findings. Furthermore, when it comes to classifying research papers, the presentation of a text is a critical stage in identifying similarities or performing statistical operations on text documents.According to the current literature, most techniques have relied on traditional statistical measures such as TF, BOW, and TFIDF, etc. The frequency of terms is usually used in these measurements to capture information. We claim that the semantics of a text should be addressed before assessing the similarity between textual documents, which has been overlooked by conventional statistical techniques. Furthermore, static threshold values have been used in multi-label classification-based techniques. In many studies, researchers have chosen to determine threshold values either by consulting domain experts or by selecting arbitrary values and ensuring them by trial and error on the dataset, which is a time-consuming procedure. We suggest that relying on domain experts or an arbitrary value is insufficient for the stated goal. We argue that a threshold value should be established based on a thorough examination of the data set in question. These problems lead to the formulation of our problem statement and its solution.

We used openly available information as a feature for the classification of research articles. We used each of these metadata separately as well as in combination. Two benchmark datasets were employed in the experiments for evaluation. The metadata from these datasets was first extracted. Title and keywords were extracted from the first dataset, and title, keywords, and General Terms were extracted from the second dataset. Following that, we created every feasible combination of these features. On both datasets, additional pre-processing is conducted, which includes tokenizing all text into words,stemming all the words into their root words, and removing all stop words and noise. Furthermore, we used a semantic model rather than using a frequency-based methodology to represent text.

The Word2Vec model captures both the semantic and contextual aspects of a term in the text. We first used a corpus of research articles to train our model. This model produces a vector space in which each word in a corpus is represented by a distinct vector. Similar word vectors are close to each other, while different word vectors are far apart. Following that, we used this trained model to convert the text in both dataset’s records to vector form.

In SLC, we simply enter the test paper into the system, and the system determines the test document’s average similarity scores with each category paper. We only have the maximum average similarity score category as a projected category against the test documents after finding scores for all of the categories.In the case of multi-label classification, we developed a method for determining threshold values for each category based on a thorough examination of datasets. Following that, we used a multi-label classification algorithm to perform multi-label classification. The system compares a test document’s average similarity score to each category paper. The average similarity scores of each category are compared to the thresholds that have already been established. As a forecast category, the category score that has reached the threshold value is chosen. When we used the metadata separately in SLC, we found that the title metadata had a higher average accuracy of 0.81 and 0.79 for the JUCS and ACM datasets, respectively. In the case of double metadata, the combination of title and keywords worked exceptionally well, with average accuracy of 0.86 and 0.84 for the JUCS and ACM datasets, respectively When we looked at the metadata separately in MLC, we found that the keywords metadata had a higher average accuracy of 0.75 and 0.73 for the JUCS and ACM datasets, respectively. In the case of double metadata, the title and keywords combination performed exceptionally well, with average f-scores of 0.81 and 0.80 for the JUCS and ACM datasets, respectively, like SLC. The JUCS dataset has no triple metadata combination, however the ACM title, keywords, and general terms combination has an average f-score of 0.77.

We compared our findings to two state-of-the-art techniques. When compared to Khor or tang methods, the single label classification results are superior. On research papers, this method used metadata as a feature and attained an average accuracy of 0.83. Our method made use of metadata as well, achieving average accuracy of 0.86 on JUCS datasets and 0.84 on ACM datasets. The results of the multi-label categorization were compared to those of Ali and Asghar. Their method also included metadata as a feature, yielding values of 0.78 and 0.77 on the JUCS and ACM datasets, respectively, while our method yielded 0.81 and 0.80 on the JUCS and ACM datasets. The overall findings of this work are that: (1) In scenarios where content is not available, we can use metadata as a replacement, which can achieve good results up to a point; (2) We have used a semantic model for text representation, which performed better than conventional statistical features; and (3) The proposed method decreases the cognitive effort necessary to define a threshold value that requires domain expertise.

## Conclusion

Classification of research articles into predefined categories is deemed as an important research problem from the past several years. An accurate classification model to label the research papers into different categories can boost the efficiency of various digital libraries. It can also assist the scholarly community by providing them content to conduct a literature review on a particular topic or domain. Critical analysis of state-of-the-art research articles classification has revealed that most of the schemes have employed the content of research articles and a few of them have harnessed the metadata to classify research papers into different categories but failed to produce promising results. Similarly, in the case of representation of text, these schemes have employed statistical measures which have ignored the semantic context of the text. Moreover, in the case of multi-label classification, while assigning multiple categories threshold values are required which is mostly provided by domain experts without knowing the nature of the dataset in existing techniques. In this study, we have presented a classification model that performed classification of research papers onto the top level of ACM categories with the help of metadata and its combinations. Moreover, in this model, we have used the Word2Vec model for the representation of text which captured the semantic context of the text. To address the problem of finding the threshold, we have proposed a method for determining threshold values for each category based on a thorough examination of datasets. The empirical results have revealed that on JUCS and ACM datasets, the proposed SLC model improves accuracy up to 4%, while the proposed MLC model increases accuracy by 3%. Moreover, we have also observed that a semantic model for text representation is better than conventional statistical features and the proposed method for finding threshold decreases the cognitive effort necessary to define a threshold value that requires domain expertise. The flaw of our model is that it’s a time-consuming learning method because we have to calculate the average similarity of each test paper with each category paper every time. Our findings would be helpful for researchers to classify articles more efficiently by overcoming the time limitation and by upgrading the classification to the next ACM taxonomy level.
